# Angiotensin-(1-7) Promotes Resolution of Neutrophilic Inflammation in a Model of Antigen-Induced Arthritis in Mice

**DOI:** 10.3389/fimmu.2017.01596

**Published:** 2017-11-20

**Authors:** Lívia C. Barroso, Giselle S. Magalhaes, Izabela Galvão, Alessandra C. Reis, Daniella G. Souza, Lirlândia P. Sousa, Robson A. S. Santos, Maria Jose Campagnole-Santos, Vanessa Pinho, Mauro Martins Teixeira

**Affiliations:** ^1^Department of Biochemistry and Immunology, Biological Sciences Institute, Federal University of Minas Gerais, Belo Horizonte, Brazil; ^2^Department of Physiology and Biophysics, Biological Sciences Institute, Federal University of Minas Gerais, Belo Horizonte, Brazil; ^3^Department of Morphology, Biological Sciences Institute, Federal University of Minas Gerais, Belo Horizonte, Brazil; ^4^Department of Microbiology, Biological Sciences Institute, Federal University of Minas Gerais, Belo Horizonte, Brazil; ^5^Department of Clinical and Toxicological Analysis, School of Pharmacy, Federal University of Minas Gerais, Belo Horizonte, Brazil

**Keywords:** resolution of inflammation, angiotensin (1-7), apoptosis, neutrophils, efferocytosis

## Abstract

Defective resolution of inflammation may be crucial for the initiation and development of chronic inflammatory diseases, such as arthritis. Therefore, it has been suggested that therapeutic strategies based on molecules that facilitate inflammation resolution present great potential for the treatment of chronic inflammatory diseases. In this study, we investigated the effects and role of angiotensin-(1-7) [Ang-(1-7)] in driving resolution of neutrophilic inflammation in a model of arthritis. For this purpose, male C57BL/6 mice were subjected to antigen-induced arthritis and treated with Ang-(1-7) at the peak of the inflammatory process. Analysis of the number of inflammatory cells, apoptosis, and immunofluorescence for NF-κB was performed in the exudate collected from the knee cavity. Neutrophil accumulation in periarticular tissue was measured by assaying myeloperoxidase activity. Apoptosis of human neutrophil after treatment with Ang-(1-7) was evaluated morphologically and by flow cytometry, and NF-κB phosphorylation by immunofluorescence. Efferocytosis was evaluated *in vivo*. Therapeutic treatment with Ang-(1-7) at the peak of inflammation promoted resolution, an effect associated with caspase-dependent neutrophils apoptosis and NF-κB inhibition. Importantly, Ang-(1-7) was also able to induce apoptosis of human neutrophils, an effect associated with NF-κB inhibition. The pro-resolving effects of Ang-(1-7) were inhibited by the Mas receptor antagonist A779. Finally, we showed that Ang-(1-7) increased the efferocytic ability of murine macrophages. Our results clearly demonstrate that Ang-(1-7) resolves neutrophilic inflammation *in vivo* acting in two key step of resolution: apoptosis of neutrophils and their removal by efferocytosis. Ang-(1-7) is a novel mediator of resolution of inflammation.

## Introduction

Rheumatoid arthritis (RA) is an inflammatory autoimmune disease characterized by chronic and deforming destructive arthritis accompanied by systemic dysfunctions (1). RA affects approximately 1% of the adult population in the word (2). The cost of RA is very high, associated with decreased productivity and impact on quality of life (3). Neutrophils are the most abundant leukocytes recruited to the joints of patients with active RA (4, 5) and can contribute to aggravate articular inflammation and tissue damage (6).

In chronic inflammatory diseases, persistent inflammation contributes to tissue damage and the consequent loss of organ function (7, 8). Persistent inflammation may be secondary to the constant influx of leukocytes due to the continued production of inflammatory mediators (9). However, recent evidences suggest that the inability to resolve ongoing inflammation may be a major contributor to persistent inflammation. Resolution of inflammation is an active process, finely tuned by mediators that (i) prevent excessive trafficking of leukocytes to the site of damage (10); (ii) shutdown intracellular signaling molecules associated with cytokine production and leukocyte survival (11); (iii) induce apoptosis of recruited inflammatory cells; and (iv) promote clearance of apoptotic cells (especially by macrophages) by a process named efferocytosis (12). Altogether, these actions will allow the cessation of inflammation and reestablishment of tissue homeostasis (13, 14). In RA, reduced neutrophil apoptosis correlate positively with disease severity (15, 16).

Angiotensin-(1-7) [Ang-(1-7)] is a peptide of the renin-angiotensin system mainly known to be involved with the control of cardiovascular, renal, and vascular functions. This peptide gained interest in recent years due to its anti-inflammatory and anti-fibrotic effects in different acute and chronic inflammatory conditions, including asthma (17), dyslipidemia (18), renal injury (19, 20), and myocardial infarction (21). Ang-(1–7) is mostly synthesized by ACE2 (22, 23), and the majority of its biological actions are mediated through the G-coupled Mas receptor (24), counterbalancing the effects of the ECA/Ang II/AT1 receptor pathway in different pathophysiology processes.

In previous studies, our group demonstrated that preventive administration of Ang-(1-7) or a synthetic analog, AVE0991, decreased rolling and adhesion of leukocytes to the microvascular endothelium at inflamed joints in a model of antigen-induced arthritis (AIA) (25). The inhibition of leukocyte interaction with endothelial cells was associated with decreased neutrophil influx into the joints, cytokine release inhibition, and improvement of joint hypernociception (25). In this study, we investigated the effects of therapeutic administration of Ang-(1-7) in driving resolution of neutrophilic inflammation in a model of arthritis. Our results demonstrate marked and novel pro-resolving actions of Ang-(1-7) *in vivo* in two key steps of the resolution process—apoptosis and efferocytosis.

## Materials and Methods

### Animals

Eight to ten weeks old male C57Bl/6 mice (20–25 g) were obtained from the animal facility of our institution. Animals were maintained under temperature-controlled condition with an artificial 12 h light–dark cycle with free access to chow and water. The study was approved by the local Animal Ethics Committee (CETEA 192/2012).

### AIA in Mice

To induce arthritis, animals were immunized with an intradermal injection of 100 µg of methylated bovine albumin (mBSA, Sigma, St. Louis, MO, USA), emulsified in 500 µg of Freund’s complete adjuvant (CFA, Sigma) at the base of the tail (day 0). Two weeks after immunization, antigen challenge was performed by injection of 10 µg of mBSA diluted in 10 µl of sterile saline into the left joint. As control group received intra-articular injection of PBS (10 µl) into the same site. Mice were treated with an intra-articular injection (10 µl) of 100 ng of Ang-(1-7) (Bachem, Torrance, CA, USA), Mas antagonist, A779 [D-Ala^7^-Ang-(1-7), 200 ng/cavity; Bachem, Torrance, CA, USA], or the vehicle (NaCl 0.9%) 12 h after antigen challenge (26). All surgical procedures were performed under ketamine and xylazyne anesthesia (150 and 10 mg/kg, respectively) followed by euthanasia.

### Tissue Neutrophil Infiltration

The extent of neutrophil accumulation in periarticular tissue was measured by assaying myeloperoxidase (MPO) activity, as described elsewhere (27). Results were expressed as the relative number of neutrophils per milligram of synovial tissue. Intra-articular neutrophil infiltration 24 h after antigen challenge was measured in the liquid collected from the knee cavity after been washed twice with 10 µl BSA. The total number of leukocytes was counted in Neubauer chamber after staining with Turk’s solution. Differential leukocyte counts were obtained after staining of Cytospin slides with May–Grünwald–Giemsa using standard morphologic criteria.

### Calculation of Resolution Indices

We quantified the resolution indices as described (28, 29). Murine synovial fluid was collected at 12, 24, 36, 48, and 72 h after challenge with mBSA. The treatment with Ang-(1-7) was performed at the peak of inflammation, 12 h after the challenge. The number of PMN and mononuclear cells was determined by total and differential leukocyte counting. The resolution of acute inflammation were defined in quantitative terms by the following resolution indices: (1) magnitude (ψ_max_ and *T*_max_), ψ_max_ (maximal PMN), *T*_max_ (time point when PMN numbers reach maximum); (2) duration (*T*_50_), *T*_50_ (time point when PMN numbers reduce to 50% of maximum); and (3) and *R_i_* (the interval between *T*_max_ and *T*_50_, when 50% of PMNs are lost from the synovial fluid) (28).

### Assessment of Leukocyte Apoptosis

Apoptosis was assessed morphologically, as reported previously (13, 30, 31). Briefly, 5 × 10^4^ cells collected 24 h after antigen challenge were cyto-centrifuged, fixed and stained with May–Grünwald–Giemsa. Cells were counted using oil immersion microscopy (×100 objective) to determine the proportion of cells with distinctive apoptotic morphology, i.e., cells that presented chromatin condensation, nuclear fragmentation, and formation of apoptotic bodies, both out or inside macrophages. At least 500 cells were counted/slide and the results expressed as percentage of cells with apoptotic morphology. Assessment of apoptosis was also performed by flow cytometry using FITC-labeled Annexin-V (ApoDETECT Annexin V-FITC kit; Invitrogen, Carlsbad, CA, USA) and PI as an index of loss of nuclear membrane integrity.

### Isolation of Neutrophils by Density Gradient Centrifugation

Blood from healthy human donors (Ethics Committee of the Universidade Federal de Minas Gerais, Brazil—Institutional Review Board Project number 0319.0.203.000-11) was collected in EDTA tubes, and diluted 1:1 with RPMI. Human neutrophils (PMNs) were separated by gradient centrifugation over Histopaque-1119 and Histopaque-1077 (Sigma-Aldrich^®^). To avoid mixing the two densities, which will preclude cell separation during centrifugation, blood was centrifuged for 30 min at 2,000 rpm at 25°C without brake. Neutrophils were collected at the interface of the Histopaque-1119 and Histopaque-1077 layers. After wash with PBS, viable cells were counted. Neutrophils were >95% viable and >5% pure.

### Confocal Microscopy

Cells of joint lavage or PMN were centrifuged, at 1,200 rpm for 5 min at 4°C, and the pellet was resuspended in PBS, and total cell counts were performed. From the total number were taken 5 × 10^5^ cells to perform cytocentrifugation (Cytospin; Shandon Lipshaw Inc., Pittsburgh, PA, USA) in cells cover slips. Next, cells were fixed with 4% paraformaldehyde for 15 min and washed three times. Fc Block (CD16/32, BD Biosciences) was added for 30 min to block unspecific binding of antibody. To perform intracellular staining cells coverslips were permeabilized for 30 min (Perm Wash, BD Bioscience; 1:12 in PBS-BSA 1%) and incubated with the antibody overnight. For extracellular staining, antibodies were added. Finally, the coverslips were prepared with Fluoromount (Aldrich Sigma, USA) for analysis. Images were obtained in a Nikon Eclipse Ti microscope with laser confocal C2, equipped with three different lasers (excitation 405, 488, and 543 nm) and emission filter 450/50 nm (channel 1), 515/30 nm (channel 2), and 584/50 nm (channel 3). The fluorescence intensity was measured using a 6.3 Volocity software (Perkin-Elmer, USA), and the fluorescence profile was assessed using Image J (NIH, USA). The antibodies used were p65 Alexa Fluor 488 (Cell signaling; 1:100), DAPI (Cell signaling; 1:100), Siglec-F (Santa Cruz, CA, USA; 1: 100) and MAS (Alomone, 1:100) diluted in permeabilization solution (wash-perm; BD Bioscience).

### Efferocytosis Assay *In Vivo*

Human neutrophils (PMNs) were isolated from peripheral venous blood drawn from healthy volunteers, after informed written consent, as described previously (32). Briefly, PMNs were separated as described earlier and plated at 5 × 10^6^ cells/well. Apoptosis (>80%) was induced by staurosporine (10 µM), Ang-(1-7) (100 nM) or staurosporine (10 µM), and Ang-(1-7) (100 nM) by culturing the neutrophils in complete RPMI 1640 for 1 h at 37°C in 5% CO_2_ atmosphere. 4 × 10^6^ apoptotic PMNs were injected intraperitoneally into mice bearing a 72 h peritonitis elicited by 0.1 mg of zymosan. When indicated, mice were treated with different doses of Ang-(1-7) (0.1–3.0 μg/mice) 30 min before the injection of apoptotic cells. Animals were killed after 90 min later, and exudates were collected for morphologic analysis (33, 34).

### Data Statistical Analysis

All results are expressed as mean ± SEM. Comparisons among three or more groups were performed by one-way ANOVA followed by Newman–Keuls *post hoc* test. Significance between two groups was assessed by Student’s *t*-test. All analysis and graphics were performed with GraphPad Prism software (version 5.0; La Jolla, CA, USA). The level of significance was set to *p* ≤ 0.05.

## Results

### Ang-(1-7) Resolves Neutrophilic Inflammation in Arthritis

#### Ang-(1-7) Promotes Resolution by Inducing Neutrophil Apoptosis

**Figure 1:**
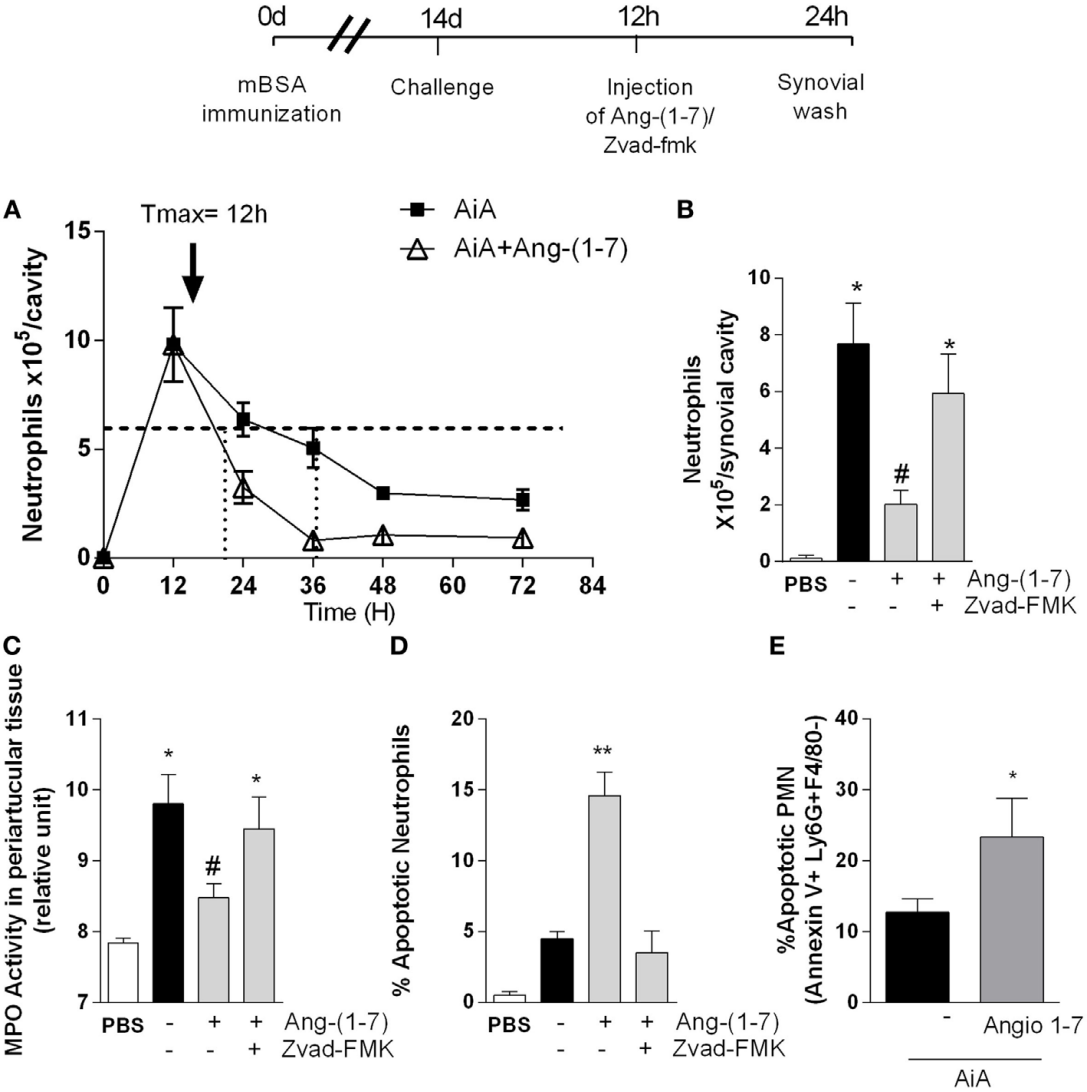
Angiotensin-(1-7) [Ang-(1-7)] promotes resolution of the inflammatory response by inducing neutrophils apoptosis in a model of antigen-induced arthritis (AIA) in mice. **(A)** Experimental protocol: on day 0, mice were immunized by injecting 10 µg of mBSA into each knee joint. Fourteen days later, arthritis was induced by an additional injection of 100 µg of mBSA into each joint (Challenge). At the peak of inflammation (12 h post-mBSA challenge, arrow), 100 ng of Ang-(1-7) was injected in mice knee joints. Some animals also received 1 mg/kg of the caspase inhibitor, zVAD-FMK 10 min before Ang-(1-7) treatment. Control mice were injected with 10 µL of PBS. At 24 h post-challenge (12 h after treatment), the mice joints were washed; **(A)** the number of neutrophils measured at different time points 12, 24, 36, 48, and 72 h post-arthritis induction and the calculated kinetics parameters and resolution index; **(B)** number of neutrophils present in the synovial cavity; **(C)** relative number of neutrophils present in the periarticular tissue, as determined by the activity of myeloperoxidase (MPO); **(D)** percentage of apoptotic neutrophils 24 h post-arthritis induction; **(E)** the increase in the percentage of apoptotic neutrophils evaluating the expression of Annexin-V on the surface of neutrophils. Scale = 10 µm. Bars show the mean ± SEM from eight mice per group. *p ≤ 0.05 when compared with PBS-treated group; ^#^*p* ≤ 0.05 when compared with AIA group. Differences between groups were evaluated by ANOVA, followed by a Student–Newman–Keuls test.

We used a well-established model of AIA in mice (Figure 1) to evaluate the effect of Ang-(1-7) on inflammatory responses (26) and resolution (28). In this model of AIA, injection of antigen in the knee joints of immunized C57BL/6 mice induced significant neutrophil influx that peaked from 12 to 24 h after the challenge with mBSA (15). We have previously shown that treatment with Ang-(1-7) before antigen challenge prevented neutrophil influx (25). Herein, we investigated the effect of local administration of Ang-(1-7) on the kinetics of neutrophil influx in AIA. Mice received challenge injection of mBSA, and 12 h later, an articular Ang-(1-7) injection, and cells were collected at 12, 24, 36, 48, and 72 h after challenge. The treatment of mice with Ang-(1-7) shortened *R_i_*: *R_i_*_AIA_ ~ 25 h; *R_i_*_Ang-(1-7)_ ~ 9 h. Therefore, Ang-(1-7) treatment given 12 h after antigen challenge anticipated inflammatory resolution by 16 h (Figure 1A). In addition, the treatment with Ang-(1-7) at the peak of the inflammatory response decreased neutrophil accumulation in the synovial cavity (Figure 1B) and in periarticular tissues (Figure 1C). There was also increase in the percentage of apoptotic neutrophils (Figure 1D). The increase in the percentage of apoptotic neutrophils was confirmed by experiments evaluating the expression of Annexin-V on the surface of neutrophils (Figure 1E).

Systemic administration of Ang-(1-7) (Figures S1A,D in Supplementary Material) as an inclusion compound containing Ang-(1-7) [HPβCD-Ang-(1-7); Figures S1B,E in Supplementary Material] or local treatment with Ang-(1-7) (Figures S1C,F in Supplementary Material) at the peak of inflammation decreased the number of neutrophils in the synovial cavity and periarticular tissues. Both local and systemic Ang-(1-7) administration were equally effective and maximal inhibition occurred at 100 ng/cavity. Consistent with the latter findings, administration of the broad-spectrum caspase inhibitor, zVAD-FMK, prevented the ability of Ang-(1-7) to resolve neutrophilic inflammation and to induce apoptosis in this AIA model (Figures 1B–D). These data suggest that Ang-(1-7) promotes resolution of the inflammation by inducing caspase-dependent neutrophil apoptosis. A similar reduction was not observed in mononuclear cells (data not shown).

#### The Effects of Ang-(1-7) Are Mas-Dependent

**Figure 2:**
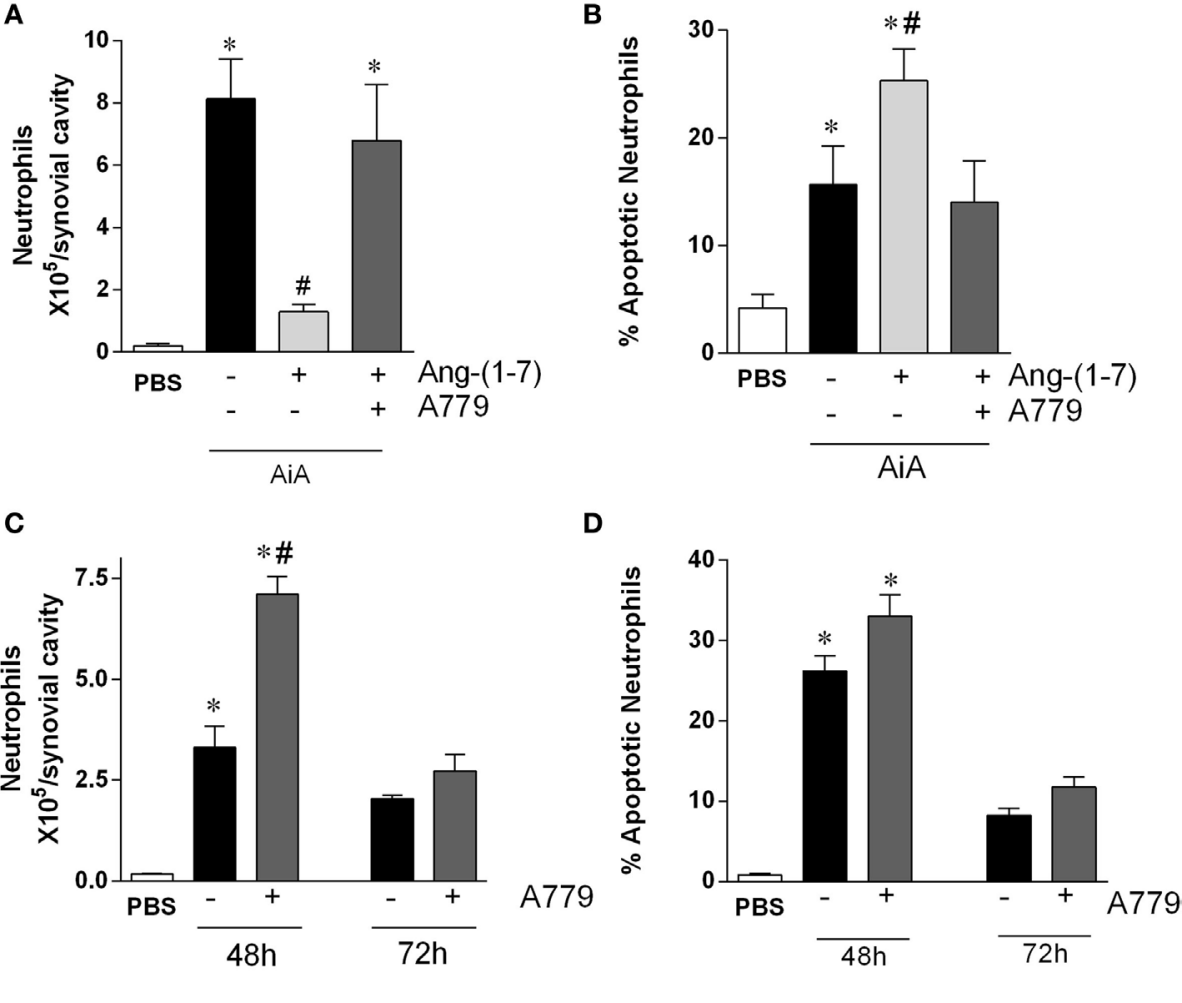
The Mas antagonist, A779, reverses the pro-resolution effect of angiotensin-(1-7) [Ang-(1-7)] in a model of arthritis in mice. **(A,B)** The number of neutrophils and the percentage of apoptotic neutrophils in the synovial cavity, respectively, in antigen-induced arthritis (AIA) mice 12 h after treatment with Ang-(1-7) alone or Ang-(1-7) and A779. **(C,D)** The number of neutrophils and the percentage of apoptotic neutrophils in the synovial cavity, respectively, in AIA mice 48 and 72 h after challenge treated with A779. Bars show the mean ± SEM from eight mice per group. *p ≤ 0.05 as compared with PBS-control group; ^#^*p* ≤ 0.05 as compared with AIA group (one-way ANOVA followed by Newman–Keuls test).

Next, we investigated the role of the *Mas* receptor in mediating the pro-resolving activity of Ang-(1-7). In agreement with data shown in Figure 1, administration of Ang-(1-7) 12 h after antigen challenge decreased inflammation and promoted resolution of inflammation by decreasing neutrophil accumulation in the synovial cavity (Figure 2A) and increasing the percentage of neutrophil apoptosis (Figure 2B). Blockade of the Mas receptor by treatment with a selective receptor antagonist, A779, prevented the pro-resolving actions of Ang-(1-7) (Figures 2A,B).

We then assessed whether Ang-(1-7) had an endogenous effect on the resolution of AIA. To this end, we treated animals with A779 at 12 h after antigen challenge and natural resolution of neutrophil inflammation was followed till 72 h after challenge. As it can be seen in Figure 2C, the number of neutrophils in the joint was greater in mice treated with A779 at 48 h, but not 72 h, after challenge. Therefore, A779 was able to delay the kinetics of natural resolution of AIA, but eventually resolution occurred both in control and drug-treated mice (Figure 2C). There were no significant changes in number of apoptotic neutrophils at the time points examined (Figure 2D).

#### Ang-(1-7) Induced Apoptosis in Human Neutrophils

**Figure 3:**
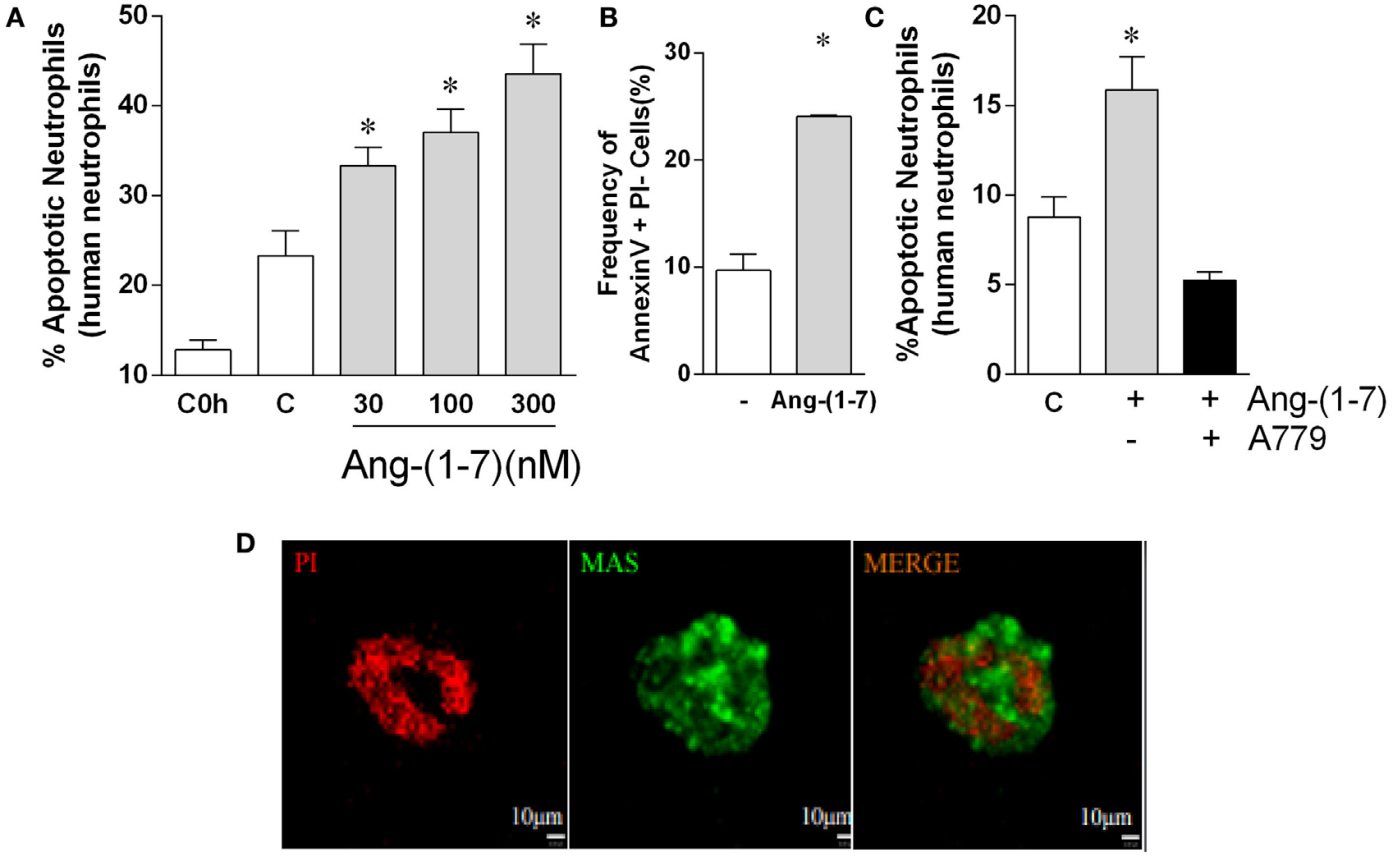
Angiotensin-(1-7) [Ang-(1-7)] induces apoptosis of human neutrophils. Ang-(1-7) (30, 100, and 300 nM/well) or PBS (control, C) was incubated for 4 h with isolated human neutrophils. A779 (300 nM/well) was added 15 min before Ang-(1-7). **(A)** Percentage of apoptotic human neutrophils in response to increasing concentration of Ang-(1-7); **(B)** percentage of cells expressing Annexin V after 4 h incubation with Ang-(1-7) (100 nM/well); **(C)** effect of the Mas antagonist, A779, on neutrophil apoptosis induced by Ang-(1-7); **(D)** images of neutrophils stained for Mas receptor (green) and propidium iodide (PI, red). *p ≤ 0.05 when compared with PBS control (one-way ANOVA followed by Newman–Keuls test).

Next, we explored whether Ang-(1-7) would also induce apoptosis in human neutrophils. Neutrophils undergo quick spontaneous apoptosis *in vitro* (Figure 3). Treatment with Ang-(1-7) induced a concentration-dependent increase in spontaneous apoptosis of human neutrophils, as assessed by morphological criteria (Figure 3A). Similar results were obtained when neutrophil apoptosis was analyzed by flow cytometry (Figure 3B). The effects of Ang-(1-7) were Mas receptor-dependent, as seen by the inhibitory effect of A779 on Ang-(1-7)-induced neutrophil apoptosis (Figure 3C). Since the cells responded to Ang-(1-7), we investigated whether these cells expressed the *Mas* receptor. Using real time-PCR, we observed significant *Mas* mRNA expression in human neutrophils (Figure S2 in Supplementary Material). Mas expression on human neutrophils was confirmed by immunofluorescence (Figure 3D).

#### Ang-(1-7)-Induced Resolution *In Vivo* and Apoptosis of Human Neutrophils *In Vitro* Are Associated with Decreased Phosphorylation of p65 NF-κB

**Figure 4:**
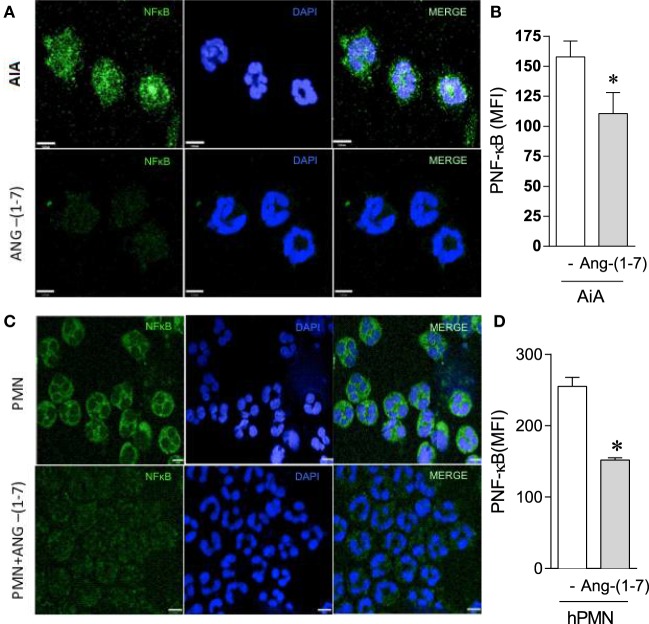
Angiotensin-(1-7) [Ang-(1-7)] decreases NF-κB activation. **(A,B)** NF-κB expression by neutrophils obtained from the knees of antigen-induced arthritis (AIA) mice treated or not with Ang-(1-7); **(C,D)** NF-κB expression by human neutrophils treated or not with Ang-(1-7). Bars show the mean ± SEM from eight mice per group. *p ≤ 0.05 when compared with PBS control (one-way ANOVA followed by Newman–Keuls test).

The transcription factor NF-κB is known to contribute to the survival of neutrophils *in vivo* (35). As it can be seen in Figure 4, treatment with Ang-(1-7) was associated with decrease in NF-κB activation in neutrophils recruited to the synovial cavity of the mice, as seen by the decreased phosphorylation of this transcription factor (Figures 4A,B). Interestingly, treatment of human neutrophils with Ang-(1-7) decreased NF-κB activation (Figures 4C,D), an important survival factor for neutrophils (35). IkBα was evaluated by western blot to confirm the previous data. As seen in Figure S3 in Supplementary Material, treatment of human neutrophils with Ang-(1-7) for 2 h significantly decreased pIkBα expression. These results suggest that inhibition of NF-κB phosphorylation is a major target for the ability of Ang-(1-7) to induced apoptosis of neutrophils.

#### Ang-(1-7) Increases the Efferocytic Ability of Murine Macrophage

**Figure 5:**
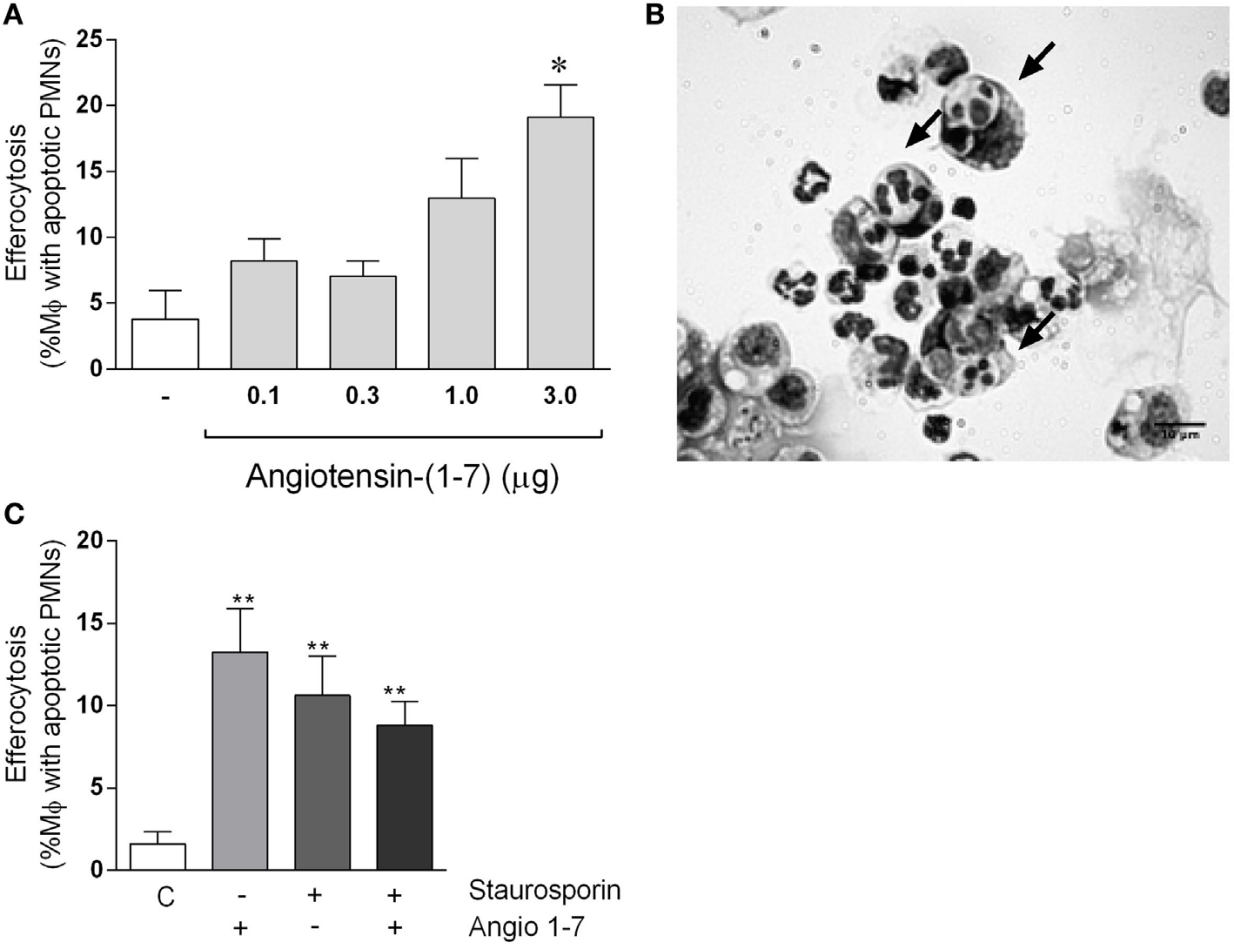
Angiotensin-(1-7) [Ang-(1-7)] induces efferocytosis of apoptotic human neutrophils. In panel **(A)**, human neutrophils (PMNs) were isolated from peripheral venous blood and apoptosis induced by staurosporine (10 µM). Apoptotic PMNs were injected intraperitoneally into mice bearing a 72-h peritonitis elicited by 0.1 mg of zymosan; these mice were injected previously (30 min before apoptotic cells) with Ang-(1-7) (0.1, 0.3, 1.0, and 3.0 μg/mice). Mice were killed after 90 min, and exudates were collected for morphologic analysis; in panel **(B)**, an image showing an example of efferocytosis of apoptotic neutrophils by macrophages. In panel **(C)**, human PMNs were treated with Ang-(1-7), staurosporine, or both before injection intraperitoneally into mice bearing a 72-h peritonitis elicited by 0.1 mg of zymosan. Mice were killed after 90 min, and exudates were collected for morphologic analysis. In panels **(A,C)**, human neutrophils were obtained from a pool of three donors, and there were five mice in each group.

Leukocyte apoptosis and subsequent removal of apoptotic cells by phagocytes (efferocytosis) are crucial events for the resolution of inflammation (36). Our data showed that Ang-(1-7) promotes resolution by inducing leukocyte apoptosis. Next, experiments were performed to investigate the ability of Ang-(1-7) to regulate macrophage efferocytosis. To this end, apoptotic cells were injected in the peritoneal cavity of mice, their engulfment by zymosan-recruited peritoneal macrophages evaluated and presented as an efferocytosis index (33, 37, 38). At the time of injection, over 90% of neutrophils were apoptotic. In addition, at 72 h after zymosan injection, macrophages were the very predominant cell type in cavity. These results are in agreement with previous studies in the laboratory (38). Efferocytosis of apoptotic neutrophils by resident macrophages was evaluated 90 min after injection of apoptotic neutrophils in the mice peritoneal cavity of mice. Figure 5A shows that treatment with Ang-(1-7) (0.1, 0.3, 1, or 3 µg) significantly increased efferocytosis of apoptotic human neutrophils. Figure 5B shows representative images of efferocytosis in macrophage stimulated with Ang-(1-7). We also tested whether treatment of neutrophils themselves would modify their subsequent efferocytosis by macrophages. To this end, human neutrophils were treated with Ang-(1-7) (100 nM), staurosporine (10 µM), or both for 1 h, and efferocytosis of apoptotic neutrophils by resident macrophages evaluated 90 min after injection of apoptotic neutrophils. As seen in Figure 5C, treatment of neutrophils with Ang-(1-7) also increased their subsequent uptake by macrophages.

## Discussion

Resolution of inflammation is an active process that tempers inflammation. Resolution is associated with increased neutrophil apoptosis and their timely removal by macrophages and aims to restore tissue homeostasis (39). Defective resolution of inflammation is thought to be crucial for the initiation and development of chronic inflammatory diseases, including RA (15, 25). Therefore, it has been suggested that therapeutic strategies based on mechanisms or molecules that facilitate inflammation resolution present great potential for the treatment of chronic inflammatory diseases. In this study, we evaluated the effects and therapeutic potential of Ang-(1-7) administration in the context of inflammatory resolution in an animal model of arthritis. We demonstrated that treatment with Ang-(1-7) at the peak of inflammation, i.e., after the influx of cells: (i) reduced the accumulation of neutrophils in inflammatory sites; (ii) induced apoptosis of neutrophils; and (iii) increased macrophage efferocytosis, which is consistent with its pro-resolving activities. We also showed that the (iv) pro-resolving effects of Ang-(1-7) were Mas receptor dependent, as demonstrated by experiments with the Mas receptor antagonist, A779. Further, we showed that (v) Ang-(1-7) induced apoptosis of human neutrophils *in vitro*, and the resolution of neutrophilic inflammation was associated with a decrease of NF-κB phosphorylation. Altogether, these results demonstrate for the first time a marked pro-resolving action of Ang-(1-7) in a model of neutrophilic inflammation *in vivo*.

Previous studies have shown that preventive administration of Ang-(1-7) decreased inflammatory responses in various models of inflammation, including arthritis (17, 20, 25). The anti-inflammatory effects of Ang-(1-7) were Mas receptor-dependent and decreased inflammatory responses through the inhibition of leukocyte migration and cytokine expression (19, 40). Here, we demonstrate that therapeutic administration of Ang-(1-7), i.e., administration of Ang-(1-7) when inflammatory responses had already been established, resolved inflammation by inducing apoptosis and efferocytosis of leukocytes. Indeed, Ang-(1-7) resolved neutrophilic inflammation and induced apoptosis of neutrophils in a model of AIA. The effects of Ang-(1-7) were Mas receptor dependent and were followed by inhibition of NF-κB and caspase activation. Of note, we could observe the therapeutic action of Ang-(1-7) when it was given locally or systemically. Indeed, pro-resolving actions were resolved when Ang-(1-7) was given as an inclusion with cyclodextrin by the oral route, suggesting that activation of this pathway may eventually be exploited for the treatment of patients with RA.

Activation of the PI3K/AKT/NF-κB axis is a major survival pathway of leukocytes, including neutrophils (15, 31). Thus, inhibiting these pathways may result in apoptosis of leukocytes and resolution of inflammation. Indeed, we have previously shown that the resolution of established inflammation was associated with the blockade of NF-κB pathway (15). Furthermore, pro-resolutive molecules, such as Annexin-A1, induce leukocyte apoptosis and resolution of inflammation *in vivo* in part by regulating the activity of NF-κB (13). Our data clearly show that Ang-(1-7) decreases phosphorylation of NF-κB pathway, an effect that may partially explain the ability of this peptide to resolve neutrophilic inflammation. These inhibitory effects of Ang-(1-7) on the NF-κB pathway have been reported by others in different systems. For example, Ang-(1-7) decreased ovalbumin-induced perivascular and peribronchial inflammation, fibrosis, and goblet cell hyper/metaplasia, an effect that was associated with inhibition of IκB-α phosphorylation (41). Similarly, infusion of Ang-(1-7) significantly reduced infarct volume, improved neurological deficits, and suppressed NF-κB activity in a model of permanent cerebral ischemia (42). In another study, the ACE2/Ang-(1-7)/Mas axis played a crucial role in preventing LPS-induced apoptosis and inflammation of PMVECs by inhibiting the JNK/NF-κB pathways (43). Further studies are underway in our laboratories to unravel downstream mechanisms by which Mas receptor activation shuts down the NF-κB pathway and consequently induces apoptosis and resolution of inflammation.

The pro-resolving effects of Ang-(1-7) administration at the peak of inflammation were clearly Mas receptor dependent, since administration of A779 antagonist reversed these effects. Our data also revealed that treatment of arthritic mice with A779 alone delayed the natural resolution of neutrophilic inflammation. Indeed, although complete resolution was eventually attained, animals treated with A779 had significantly more neutrophils in the joint than vehicle-treated animals. These findings are in keeping with previous data derived from Mas-deficient mice in which neutrophilic inflammation was prolonged (25, 44). Although further studies are warranted, our results clearly indicate that endogenous production of Ang-(1-7) and activation of Mas receptors contribute to the normal resolution of inflammation.

For resolution of inflammation to occur in an orderly fashion and for tissue homeostasis to be restored, apoptotic cells must be engulfed by tissue macrophages, a process referred to as efferocytosis. It is believed that agents that promote efferocytosis may contribute to the resolution of inflammation (45–47). For example, plasmin and plasminogen promote macrophage reprogramming and efferocytosis conduce to efficient resolution (38). Using a well-known approach to study efferocytosis *in vivo* (34, 48), our data demonstrate that treatment with Ang-(1-7) enhances apoptotic leukocyte clearance, suggesting that this effect may contribute to the pro-resolving effects of this mediator. The Mas receptor is known to be important factor for macrophage function during inflammation of the vascular system (49). However, to the best of our knowledge, this is the first demonstration that Ang-(1-7) increases apoptosis of neutrophils and efferocytosis by macrophages. Our data also showed that treatment of human neutrophils with Ang-(1-7) also lead to greater efferocytosis of apoptotic neutrophils by macrophages. The latter results suggest that part of the pro-efferocytic actions of Ang-(1-7) could be due to action of this molecule on neutrophils themselves. Further studies are necessary to see whether Ang-(1-7) may induce surface receptors on dying neutrophils that would favor their phagocytosis by macrophages. In any event, this is first study that demonstrates the direct capacity of Ang-(1-7) to induced efferocytosis by acting on macrophages or neutrophils themselves. In a dextran sulfate sodium colitis model, daily Ang-1-7 treatment induced significant apoptosis of immune cells (50). In the latter study, the effects of the Ang-(1-7) appeared to be secondary to the significant systemic anti-inflammatory action of the molecules. However, the latter data do concur with our findings. Indeed, direct induction of apoptosis on neutrophils and potentially other cell types could account for the observed effects of Ang-(1-7) in colitis (50).

At least one study (49) has shown that macrophages express the *Mas* receptor and that *Mas* receptor-deficient macrophages had an increase in gene expression of pro-inflammatory markers but a decrease in anti-inflammatory markers. Gene deficient macrophages also had enhanced migration and increased T-cell activation capacities (49). Interestingly, pharmacological Mas activation led to a shift toward M(IL-4 + IL-13)-polarized macrophages *in vitro* (49). As mentioned earlier, macrophages are crucial to efferocytose apopotic neutrophils and provide anti-inflammatory signals to tissues. Further studies are needed to investigate in detail whether Ang-(1-7) could induce polarization of macrophages toward pro-resolving phenotypes. However, it is clear that Ang-(1-7) to induce macrophage efferocytosis (this study) and are capably of reprogramming macrophages away from pro-inflammatory phenotypes (49).

In conclusion, our data show that Ang-(1-7) treatment fulfills most of the criteria devised to consider a molecule as a novel mediator of resolution of inflammation (8): (i) Ang-(1-7) reduced the accumulation of neutrophils into tissue, even after the influx of cells (shown here); (ii) Ang-(1-7) promoted apoptosis of neutrophils and increased their clearance by macrophages. Taken together with studies showing that (iii) Ang-(1-7) has well documented anti-inflammatory effects and induces polarization of macrophages away from activating phenotypes (49), our studies show that Ang-(1-7) has clear pro-resolving activities and endorse future research efforts aiming at the development of novel Ang-(1-7)-based pharmacological strategies to control, prevent, and even treat arthritis. Ang-(1-7) is a novel mediator of resolution of inflammation.

## Ethics Statement

This study was carried out in accordance with the recommendations of Ethics Committee of Federal University of Minas Gerais-Brazil (Acceptance number: 0319.0.203.000-11).

## Author Contributions

GM and LB— study conception and design, data acquisition, analysis and interpretation, and drafting and revising the manuscript; DS and LS—data analysis and interpretation and revising the manuscript; IG and AR—data acquisition, analysis and interpretation, and manuscript revision; VP and RS—study conception and design, data analysis and interpretation, and manuscript edition and revision; MC-S and MT—study conception and design, data analysis and interpretation, and drafting, editing, and revising the manuscript. All the authors approved the final version of manuscript.

## Conflict of Interest Statement

The authors declare that the research was conducted in the absence of any commercial or financial relationships that could be construed as a potential conflict of interest.

## Supplementary Material

Figure S1 Therapeutic administration of angiotensin-(1-7) decreases neutrophil accumulation in the synovial cavity and periarticular tissues in a model of antigen-induced arthritis (AIA) in mice. Experimental protocol: On day 0, mice were immunized by injecting 10 µg of mBSA into each knee joint. Fourteen days later, arthritis was induced by an additional injection of 100 µg of mBSA into each joint (Challenge). At the peak of inflammation (12 h post-mBSA challenge, arrow), Ang-(1-7) was injected in peritoneal or given to gavage. Control mice were injected with 10 µL of PBS. At 24 h after challenge (12 h after treatment) the joints were washed. **(A)** Number of neutrophils present in the synovial cavity after systemic treatment with Ang-(1-7); **(B)** number of neutrophils present in the synovial cavity after systemic treatment with compound HPβCD + Ang-(1-7); **(C)** number of neutrophils present in the synovial cavity after local treatment with Ang-(1-7); **(D)** relative number of neutrophils present in the periarticular tissue, as determined by the activity of myeloperoxidase (MPO) after systemic treatment with Ang-(1-7); **(E)** relative number of neutrophils present in the periarticular tissue, as determined by the activity of MPO after systemic treatment with compound HPβCD + Ang-(1-7); **(F)** relative number of neutrophils present in the periarticular tissue, as determined by the activity of MPO after local treatment with Ang-(1-7). Bars show the mean ± SEM from eight mice per group. **p* ≤ 0.05 when compared with PBS-treated group; ^#^*p* ≤ 0.05 when compared with AIA group. Differences between groups were evaluated by ANOVA, followed by a Student–Newman–Keuls test.

Figure S2 Expression of *Mas* receptor mRNA in human neutrophils. *Mas* receptor mRNA expression was assessed using real time-PCR. **(A)** Constitutive gene GPDH (control) amplification blot; **(B)** constitutive gene melt curve GAPDH (control); **(C)** Mas receptor amplification blot; **(D)** and Mas receptor melt curve.

Figure S3 Angiotensin-(1-7) decreases pIkBα expression. Human neutrophils were treated with Ang-(1-7) or PBS for 6 h and cells evaluated by Western blot analysis for pIkBα. For loading control, membranes were reprobed with GAPDH. Blots are representative of three independent experiments using cells from different donors.
