# Supplementary figures and images for: Angiotensin-(1-7) Promotes Resolution of Neutrophilic Inflammation in a Model of Antigen-Induced Arthritis in Mice
Source: Front Immunol. 2017 Nov 20;8:1596. doi: 10.3389/fimmu.2017.01596 (PMC5701946; doi:10.3389/fimmu.2017.01596)

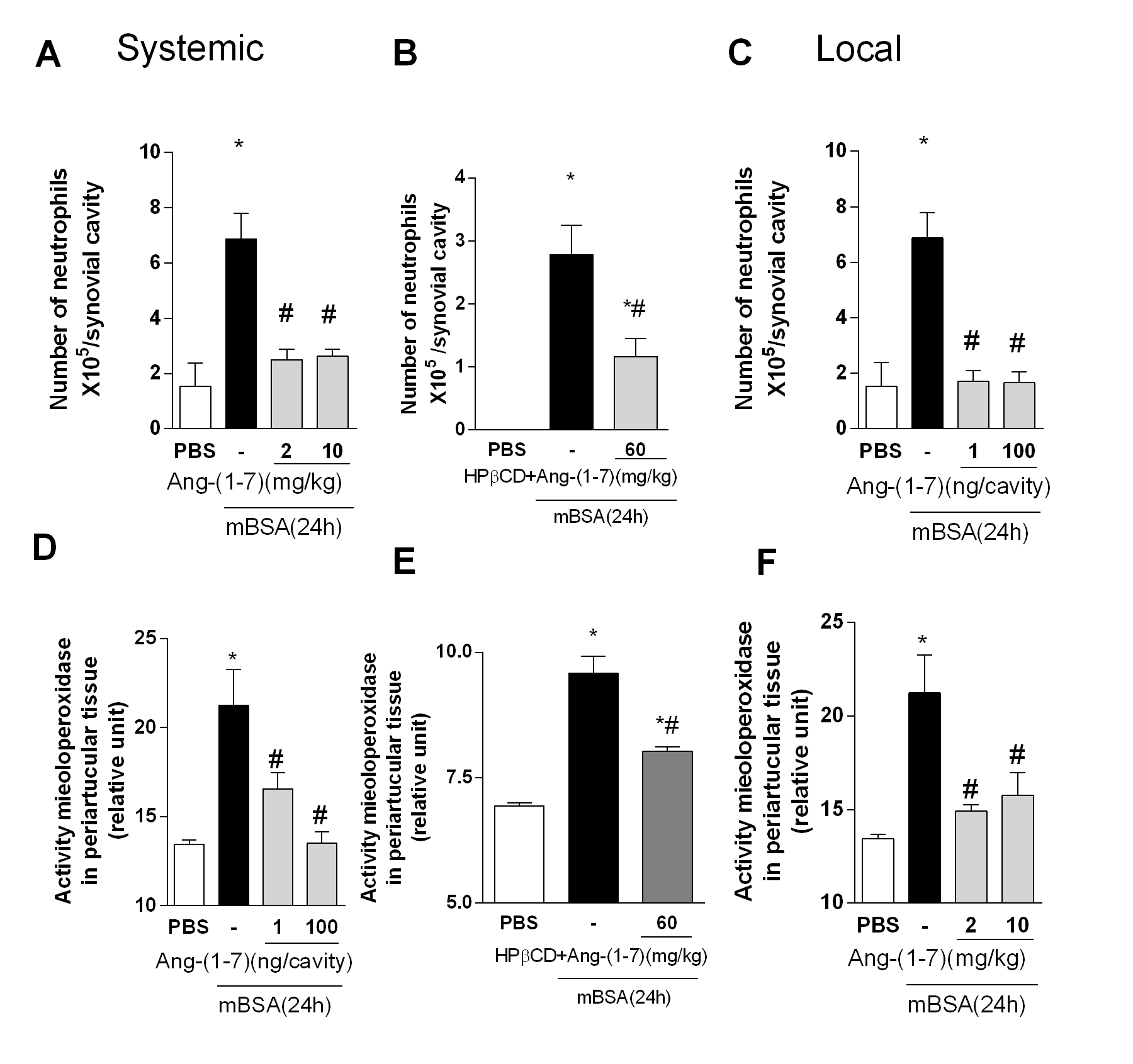

Supplement: Figure S1 — Therapeutic administration of angiotensin-(1-7) decreases neutrophil accumulation in the synovial cavity and periarticular tissues in a model of antigen-induced arthritis (AIA) in mice. Experimental protocol: On day 0, mice were immunized by injecting 10 µg of mBSA into each knee joint. Fourteen days later, arthritis was induced by an additional injection of 100 µg of mBSA into each joint (Challenge). At the peak of inflammation (12 h post-mBSA challenge, arrow), Ang-(1-7) was injected in peritoneal or given to gavage. Control mice were injected with 10 µL of PBS. At 24 h after challenge (12 h after treatment) the joints were washed. (A) Number of neutrophils present in the synovial cavity after systemic treatment with Ang-(1-7); (B) number of neutrophils present in the synovial cavity after systemic treatment with compound HPβCD + Ang-(1-7); (C) number of neutrophils present in the synovial cavity after local treatment with Ang-(1-7); (D) relative number of neutrophils present in the periarticular tissue, as determined by the activity of myeloperoxidase (MPO) after systemic treatment with Ang-(1-7); (E) relative number of neutrophils present in the periarticular tissue, as determined by the activity of MPO after systemic treatment with compound HPβCD + Ang-(1-7); (F) relative number of neutrophils present in the periarticular tissue, as determined by the activity of MPO after local treatment with Ang-(1-7). Bars show the mean ± SEM from eight mice per group. *p ≤ 0.05 when compared with PBS-treated group; #p ≤ 0.05 when compared with AIA group. Differences between groups were evaluated by ANOVA, followed by a Student–Newman–Keuls test. [file image_1.jpeg]

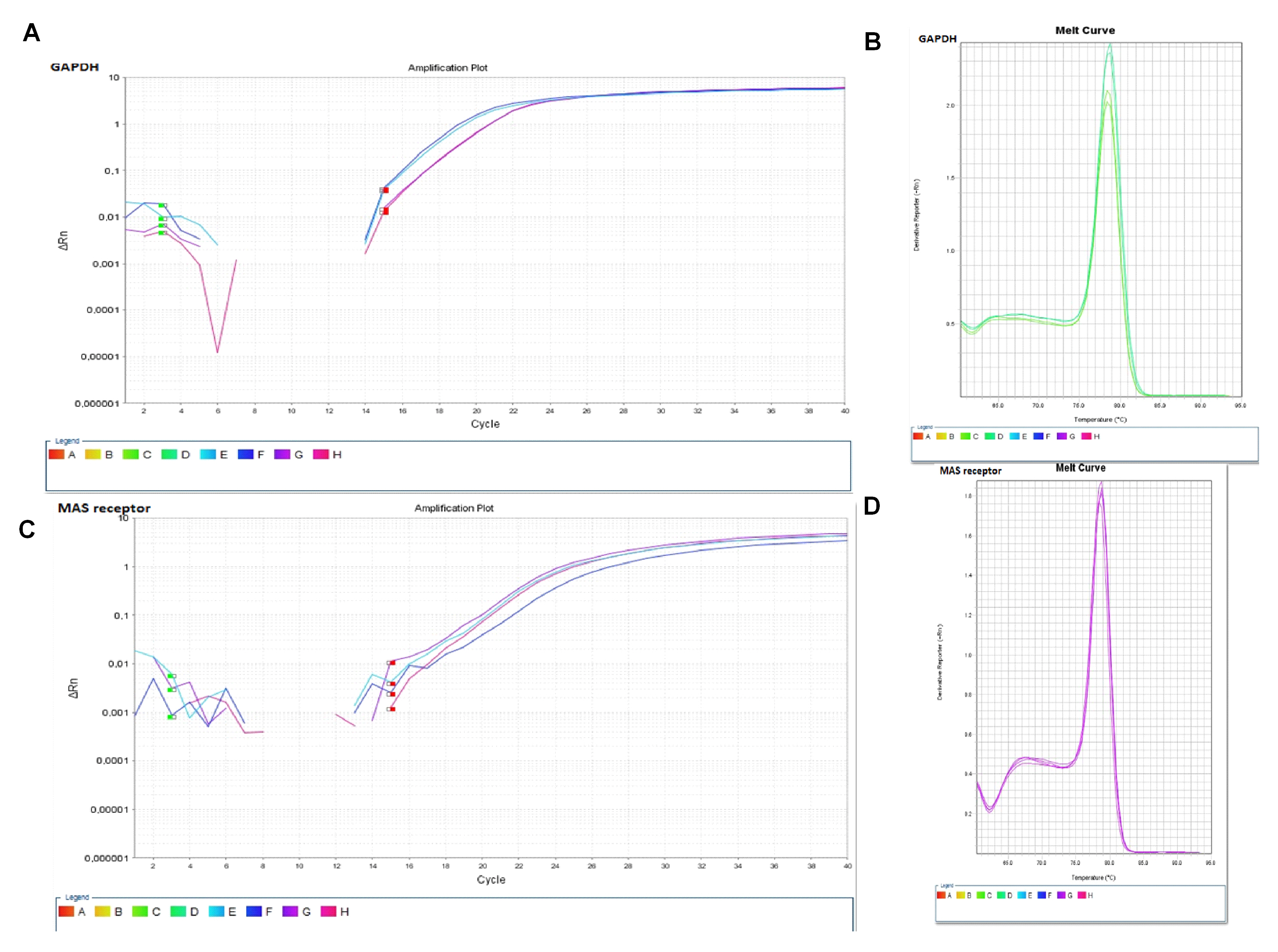

Supplement: Figure S2 — Expression of Mas receptor mRNA in human neutrophils. Mas receptor mRNA expression was assessed using real time-PCR. (A) Constitutive gene GPDH (control) amplification blot; (B) constitutive gene melt curve GAPDH (control); (C) Mas receptor amplification blot; (D) and Mas receptor melt curve. [file image_2.jpeg]

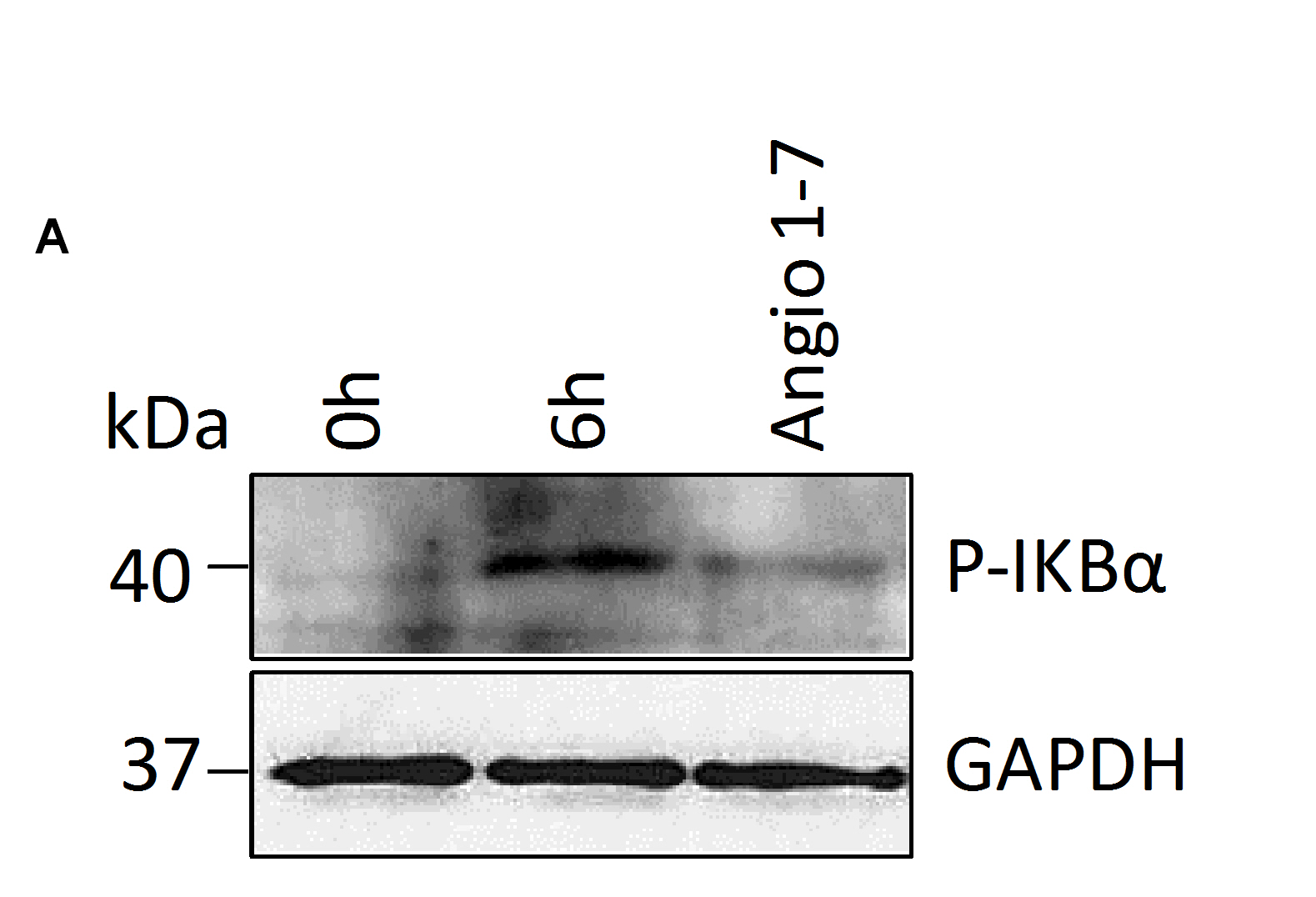

Supplement: Figure S3 — Angiotensin-(1-7) decreases pIkBα expression. Human neutrophils were treated with Ang-(1-7) or PBS for 6 h and cells evaluated by Western blot analysis for pIkBα. For loading control, membranes were reprobed with GAPDH. Blots are representative of three independent experiments using cells from different donors. [file image_3.jpeg]
